# Salivary Biomarkers for Parkinson’s Disease: A Systematic Review with Meta-Analysis

**DOI:** 10.3390/cells13040340

**Published:** 2024-02-14

**Authors:** Kacper Nijakowski, Wojciech Owecki, Jakub Jankowski, Anna Surdacka

**Affiliations:** 1 Department of Conservative Dentistry and Endodontics, Poznan University of Medical Sciences, 60-812 Poznan, Poland; annasurd@ump.edu.pl; 2 Student’s Scientific Group in Department of Conservative Dentistry and Endodontics, Poznan University of Medical Sciences, 60-812 Poznan, Poland; 86897@student.ump.edu.pl (W.O.); jjankowski41@wp.pl (J.J.)

**Keywords:** neurodegenerative diseases, Parkinson’s Disease, saliva, biomarkers, alpha-synuclein, heme oxygenase-1

## Abstract

Parkinson’s Disease (PD) is a common neurodegenerative disease which manifests with motor features, such as bradykinesia, resting tremor, rigidity, and postural instability. Using the non-invasive technique of saliva collection, we designed a systematic review to answer the question “Are salivary biomarkers reliable for the diagnosis of Parkinson’s Disease?”. Following inclusion and exclusion criteria, 30 studies were included in this systematic review (according to the PRISMA statement guidelines). Mostly proteins were reported as potential biomarkers in saliva. Based on meta-analysis, in PD patients, salivary levels of total alpha-synuclein were significantly decreased, and those of oligomeric alpha-synuclein were significantly increased. Also, according to pooled AUC, heme oxygenase-1 demonstrated significant predictive value for saliva-based PD diagnosis. In conclusion, some potential biomarkers, especially alpha-synuclein, can be altered in the saliva of PD patients, which could be reliably useful for early diagnosis of this neurodegenerative disease differentiating other synucleopathies.

## 1. Introduction

Parkinson’s Disease (PD) is one of the most common neurodegenerative disorders, with a rising global prevalence [1]. It is believed that the number of patients affected by PD increased from 2.5 million in 1990 to 6.2 million in 2015, and is estimated to reach 12.9 million by 2040 [2]. Several potential risk factors for PD have been determined: drugs, environmental toxins, male sex, genomic defects, and brain microtrauma [3]. Nevertheless, age is considered the major risk factor for this disease [4]. This remains consistent with the fact that in a population of people aged above 60 years, PD prevalence increases up to 1–2% [5].

The pathophysiology of PD includes nigrostriatal dopamine depletion and accumulation of misfolded alpha-synuclein in Lewy bodies located in the substantia nigra [6,7]. With disease progression, Lewy body pathology develops and reaches cortical and neocortical regions [8]. Currently, there is no available cure for PD; therefore, management focuses on symptomatic treatment, slowing disease development and promoting neuroprotection to achieve stability [9,10,11].

The clinical spectrum of symptoms can be divided into non-motor and motor groups [12]. Even though motor symptoms (e.g., bradykinesia, resting tremor, rigidity and postural instability) are still recognized as crucial for PD diagnosis, neuropsychiatric features (such as depression, sleep disorders and cognitive decline) are gaining similar relevance in many cases. However, non-motor symptoms may be present decades before the onset of motor symptoms [13]. Therefore, early diagnosis of PD constitutes a high challenge since there are several similar disorders resembling PD symptoms [7]. Currently, PD diagnosis is mainly based on clinical features prepared by the Movement Disorder Society, but there is a lack of specific molecular biomarkers [14,15]. Recently, progress has been made in searching for potential PD biomarkers, which might contribute to improving treatment options [16,17,18].

Saliva is considered the most non-invasive and accessible human body fluid, offering distinctive benefits over serum [19]. Furthermore, saliva collection neither requires specific skills nor is it dangerous for the medical staff or patients. Importantly, sampling can be performed several times a day, and saliva is considered a highly durable diagnostic material [20,21]. Along with numerous diagnostic advantages, saliva has been suggested as a potential biomarker for several disorders, including cancers, gastrointestinal, cardiovascular, autoimmune, and neurodegenerative diseases [22,23,24,25,26,27,28,29].

Combining beneficial properties and diagnostic values of saliva with a globally increasing problem of PD, in this systematic review, we aimed to determine the reliability of salivary biomarkers for PD diagnosis. Previous reviews considered mainly salivary alpha-synuclein as a potential marker in PD [30,31]. Therefore, focusing on only salivary origin, not the biochemical nature of biomarkers, our study was designed to answer the following question: “Are salivary biomarkers reliable for diagnosis of Parkinson’s Disease?”.

## 2. Materials and Methods

### 2.1. Search Strategy and Data Extraction

Our systematic review was conducted based on the records published from 1 January 2008 to 30 September 2023, according to the Preferred Reporting Items for Systematic Reviews and Meta-Analyses (PRISMA) statement guidelines [32], using the databases PubMed, Scopus and Web of Science. The search queries included

- for PubMed: saliva* AND (marker* OR biomarker* OR enzyme* OR metabolite* OR hormon*) AND (Parkinson* OR Alzheimer*);
- for Scopus: TITLE-ABS-KEY (saliva* AND (marker* OR biomarker* OR enzyme* OR metabolite* OR hormon*) AND (parkinson* OR alzheimer*));
- for Web of Science: TS = (saliva* AND (marker* OR biomarker* OR enzyme* OR metabolite* OR hormon*) AND (Parkinson* OR Alzheimer*)).

Retrieved search results were filtered by publication date after 1 January 2008. The search strategy deliberately included two major neurodegenerative diseases in connection with the planned publication of two separate papers. The first part about Alzheimer’s Disease has already been published recently [28].

Records were screened by the title, abstract and full text by two independent investigators. Studies included in this review matched all the predefined criteria according to PI(E)COS (“Population”, “Intervention”/”Exposure”, “Comparison”, “Outcomes” and “Study design”), as presented in Table 1. A detailed search flowchart is shown in the Section “Results”. The study protocol was registered in International prospective register of systematic reviews PROSPERO (CRD42023477115).

The results of the meta-analysis were presented in forest plots using the MedCalc Statistical Software, version 22.014 (MedCalc Software Ltd., Ostend, Belgium). The meta-analysis was performed for the most often biomarkers in saliva from patients with PD. The standardized mean differences and pooled AUC were calculated.

### 2.2. Quality Assessment and Critical Appraisal for the Systematic Review of Included Studies

The risk of bias in each individual study was assessed according to the “Study Quality Assessment Tool” issued by the National Heart, Lung, and Blood Institute within the National Institute of Health [33]. These questionnaires were answered by two independent investigators, and any disagreements were resolved by discussion between them.

**Figure 1:**
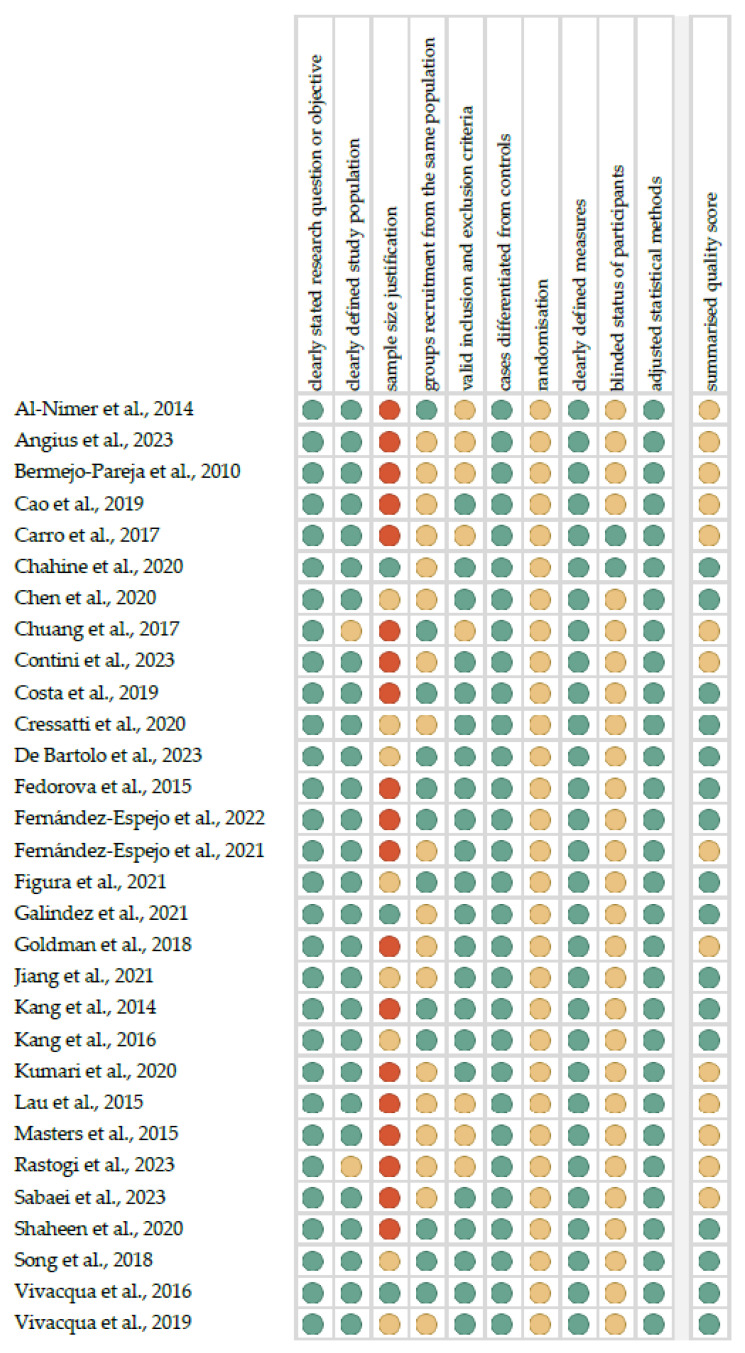
Quality assessment, including the main potential risk of bias (risk level: green—low, yellow—unspecified, and red—high; quality score: green—good, yellow—intermediate, and red—poor) [35,36,37,38,39,40,41,42,43,44,45,46,47,48,49,50,51,52,53,54,55,56,57,58,59,60,61,62,63,64].

Figure 1 shows the summarized quality assessment. The most frequently encountered risks of bias were the absence of data regarding randomization (all studies), blinding (twenty-eight studies) and sample size justification (twenty-seven studies). Critical appraisal was summarized by adding up the points for each criterion of potential risk (points: 1—low, 0.5—unspecified, 0—high). Sixteen studies (53.3%) were classified as having “good” quality (≥80% total score) and fourteen (46.7%) were classified as “intermediate” (≥60% total score).

All of the included studies had a third or fourth level of evidence (case–control studies), according to the five-grade scale the classification of the Oxford Centre for Evidence-Based Medicine levels for diagnosis [34].

## 3. Results

**Figure 2:**
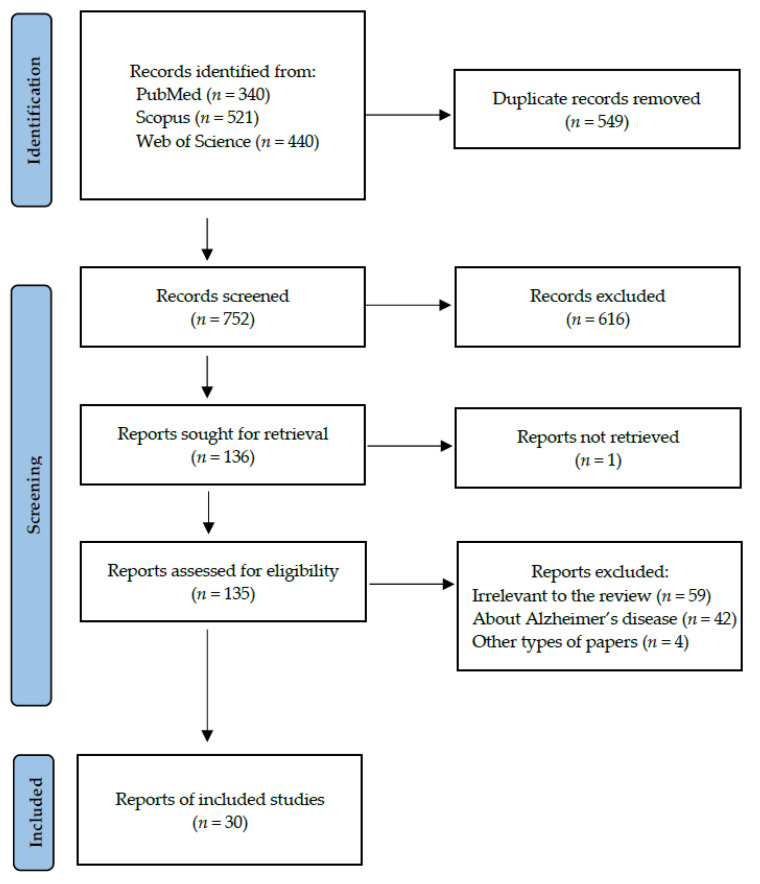
PRISMA flow diagram presenting search strategy.

Following the search criteria presented in the section “Materials and Methods”, our systematic review included thirty studies, demonstrating data collected in fourteen different countries from a total of 2032 participants with diagnosed Parkinson’s Disease. Figure 2 reports the detailed record search strategy.

In Table 2, we collected data from each eligible study included in the present systematic review, about its general characteristics, such as year of publication, setting and participants, as well as the detailed characteristics considering type of saliva, method of collection, centrifugation, storing and laboratory analysis, and potential salivary biomarkers for PD. The majority of the studies came from Europe (especially Italy). Unstimulated saliva was the most chosen diagnostic material. The researchers reported various conditions of centrifugation and storage. The most often detected potential biomarkers were proteins, determined by ELISA. Information on inclusion and exclusion criteria of study participants and their smoking status can be found in Table S1. The researchers made the PD diagnosis mainly on the basis of clinical criteria, especially according to the United Kingdom Parkinson’s Disease Society Brain Bank criteria.

Additionally, we presented the predictive parameters for most discriminant potential PD markers from included studies in Table 3. A meta-analysis was performed only for heme oxygenase-1 (HO-1), for which AUC values with confidence intervals were repeatable. The pooled AUC was 0.848 (SE ± 0.024), and HO-1 demonstrated significant predictive value for saliva-based PD diagnosis (for fixed effects, *p*-value < 0.001).

**Figure 3:**
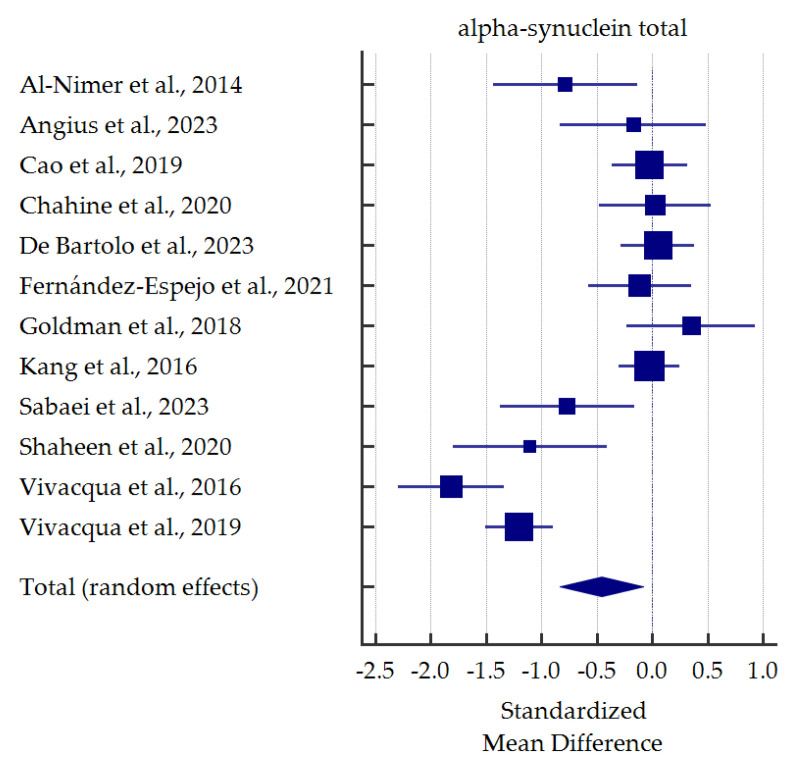
Standardized mean difference of total alpha-synuclein levels in saliva from patients with Parkinson’s Disease compared with healthy controls [35,36,37,38,40,41,42,44,45,46,47,48].

**Figure 4:**
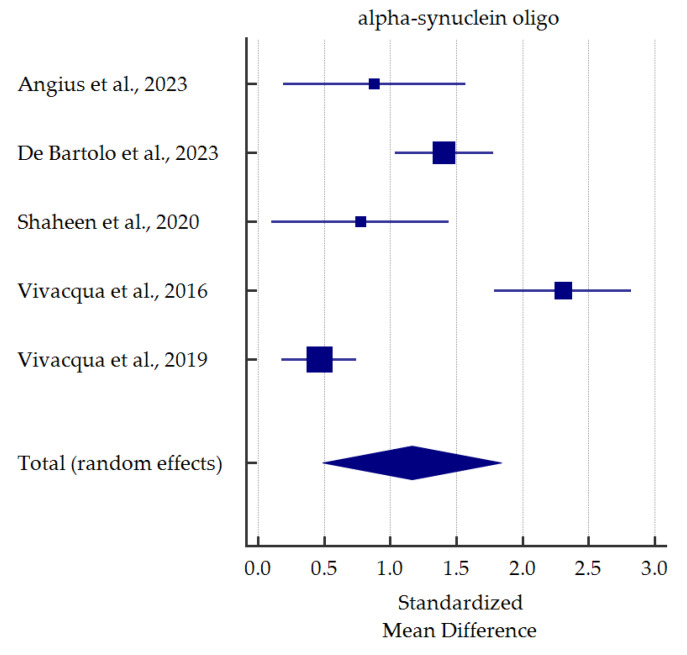
Standardized mean difference of oligomeric alpha-synuclein levels in saliva from patients with Parkinson’s Disease compared with healthy controls [36,40,45,47,48].

A meta-analysis of differences in levels between PD patients and healthy controls was conducted for alpha-synuclein (total and oligomeric forms) which was the most commonly determined biomarker in the saliva (Figure 3 and Figure 4). Salivary levels of total alpha-synuclein were significantly decreased in patients with PD. In contrast, levels of oligomeric alpha-synuclein were significantly increased in saliva of PD patients. Detailed standardized mean differences were reported in Table 4.

## 4. Discussion

### 4.1. Alpha-Synuclein

Alpha-synuclein (α-syn) is a neuronal protein concentrated in the presynaptic nerve terminals [65]. Under physiological conditions, it plays an essential role in regulating synaptic plasticity and function [66]. However, in PD pathophysiology, α-syn aggregates and forms various structures, including monomers, oligomers, and fibrils, which present different neurotoxic characteristics [67]. Indeed, evidence indicates that α-syn is not only a pathological hallmark of PD, but it might also trigger neuronal dysfunction or death [68]. α-syn toxicity may occur via several pathways, including mitochondrial or synaptic impairment, proteostasis loss, endoplasmatic reticulum stress, neuroinflammation, and aberrant cell apoptosis [69]. Interestingly, apart from the already mentioned oligomerized form of α-syn (o-α-syn), another common modification is phosphorylation at serine-129 (p-α-syn), which alters its solubility and promotes aggregation [70,71]. With the addition of total α-syn (t-α-syn), these three α-syn types have been extensively studied [72], as in studies included in this meta-analysis.

Salivary t-α-syn was analyzed in twelve studies. In five studies [35,44,45,47,48], salivary t-α-syn levels were significantly lower in PD patients than healthy controls. Moreover, Shaheen et al. [45], Vivacqua et al. [48], and Sabaei et al. [44] performed ROC analyses, which provided quite satisfactory results (AUC 0.823, not reported, 0.68; sensitivity 80.0%, 67.44%, 95.8%; specificity 86.7%, 91.04%, 36.4%;, respectively). Nevertheless, substantial discrepancies in specificity are confusing; indeed, in a study by Sabaei et al. [44], patients affected by Alzheimer’s Disease (AD) had even lower levels of t-α-syn than PD patients. On the other hand, as many as seven studies revealed no significant differences in salivary t-α-syn between the diagnostic groups [36,38,39,40,41,42,46]. Among them, most studies revealed a decreasing tendency in t-α-syn levels in PD patients. In contrast, Goldman et al. [42] and Chahine et al. [38] observed an increasing tendency. Another two studies performed ROC analyses, which did not reach satisfactory results (AUC 0.523, sensitivity 40.0 or 52.5%, specificity 51.6 or 78.3%) [36,40]. In summary, despite discrepancies in levels, salivary t-α-syn seems to be a reliable candidate for PD biomarker based on our meta-analysis. Similar findings were found in previous meta-analysis [30].

On the other hand, seven studies investigated salivary o-α-syn levels. In six studies, salivary o-α-syn levels were significantly elevated in PD groups compared to healthy controls [36,40,45,46,47,48]. Some authors performed ROC analyses of o-α-syn as a potential biomarker of PD [36,40,45,48]. The results reached a broad spectrum of diversity ranging from excellent (AUC 0.998, sensitivity 100%, specificity 98.4% [40]) up to relatively satisfactory (AUC 0.724 [45], sensitivity 57% [48], specificity 60% [36], data from different studies). In contrast, Cressatti et al. [39] presented different conclusions. In a relatively small group (22 PD patients, 30 healthy controls), salivary o-α-syn was slightly increased in the experimental group compared to controls. Nevertheless, since six out of seven studies stated a significant increase in o-α-syn, it seems it could be used as a biomarker for PD, as confirmed in our meta-analysis and previous one [30].

P-α-syn was analyzed by only one study included in this systematic review. Angius et al. [36] enrolled 15 patients and 23 healthy controls. P-α-syn levels were comparable between groups, whereas both sensitivity and specificity did not reach satisfactory results (53.33% and 78.26%, respectively). These results rather disqualify p-α-syn as a biomarker for PD; nevertheless, since the study sample was small, further investigation is necessary.

Several studies investigated ratios of α-syn forms. Angius et al. [36] investigated all three forms of α-syn. Interestingly, the p-α-syn/t-α-syn and p-α-syn/o-α-syn ratios were significantly decreased, whereas the o-α-syn/t-α-syn ratio was significantly increased in PD patients compared to healthy controls. The latter finding is consistent with the results of other studies [45,47,48]. On the other hand, interesting conclusions were presented by Kang et al. [46] in a substantial sample of 201 PD patients. The authors observed a significant decrease in the o-α-syn/t-α-syn ratio in Hoehn and Yahr stage I (H&Y stage I) PD patients, whereas patients in the II-IV stage had this ratio significantly increased. Moreover, a rising tendency within the H&Y stage was noticed, which suggested o-α-syn as a possible biomarker of PD progression.

In contrast, Shaheen et al. [45] found no statistically significant correlation between the H&Y stage and the o-α-syn/t-α-syn ratio; however, a modified H&Y scale was used [73]. Moreover, ROC analysis of the o-α-syn/t-α-syn ratio was performed in two studies (sensitivity 66.7% or 69.8%, specificity 69.6% or 95.2%, respectively [36,48]). Angius et al. [36] also presented ROC for the p-α-syn/t-α-syn and p-α-syn/o-α-syn ratios (sensitivity 46.7%, 86.7%; specificity 91.3%, 65.2%, respectively). Slightly different results in these studies need further investigation to thoroughly verify α-syn ratios’ reliability for PD diagnosis.

Interestingly, two studies that were not included were first to investigate salivary α-syn seeding activity using real-time quaking-induced conversion (RT-QuIC), an ultrasensitive seeding assay. In a brief report by Luan et al. [74], 75 PD patients, 18 multiple system atrophy (MSA) patients, and 36 healthy controls were enrolled. The results indicate that analysis of salivary α-syn based on RT-QuIC assay can be used to distinguish between PD patients and healthy participants with a satisfactory diagnostic accuracy (AUC 0.914, sensitivity 76.0%, specificity 94.4%). Moreover, significant differences in the lag phases of RT-QuIC from patients affected by PD and MSA might be used to discriminate between these disorders. Consistent results were described in a letter by Vivacqua et al. [75], in which analogical analysis was performed in a sample approximately two times smaller than in the previous study. The seeding capacity of salivary α-syn was greater in the PD group compared to healthy participants. Furthermore, the authors observed good diagnostic accuracy of salivary α-syn using RT-QuIC in distinguishing between PD patients and healthy subjects (AUC 0.844, sensitivity 83.8%, specificity 82.6%). In addition, substantial response in the salivary RT-QuIC assay significantly correlated with more severe disease stage.

Two studies investigated various α-syn forms in salivary extracellular vesicles, which will be discussed later.

### 4.2. Heme Oxygenase-1 (HO-1)

Heme oxygenase-1 (HO-1) enables heme degradation to bilirubin or biliverdin, carbon monoxide, and free iron [76]. Although it might exhibit neuroprotective properties, alterations in heme degradation lead to neurodegeneration, present in PD [77]. Moreover, studies suggest that HO-1 dysregulation is associated with neuroinflammation. Indeed, the dual role of HO-1 is not fully explained; one of the reasons might be different signaling pathways [78]. Also, it was indicated that HMOX1 gene variants could be associated to the risk of developing some forms of PD [79].

In a study by Song et al. [56], 58 PD patients and 59 healthy controls were enrolled. Salivary HO-1 levels were significantly higher in the PD group compared to controls. In further analysis, HO-1 was investigated concerning the progression of PD. Only two comparisons revealed significant differences: patients in the H&Y I stage with the H&Y III stage and patients in the H&Y I stage compared with controls. The ROC analysis showed relatively satisfactory results in distinguishing between PD patients in the early stage of the disease (H&Y I) and healthy subjects (AUC 0.76, sensitivity 75%, specificity 70%).

Another research published three years later confirmed significantly elevated levels of HO-1 in PD patients compared to healthy subjects. In this study, apart from 75 PD participants, patients with various neurological diseases were also recruited. Interestingly, although salivary HO-1 significantly differed between PD patients and patients affected by non-degenerative neurological disorders or healthy participants, the difference disappeared in comparison with AD and MCI, combined together in the degenerative group. As for disease progression, the only significant correlation was observed between non-PD controls and H&Y II stage PD patients; the comparison of non-PD controls and H&Y I stage patients reached a *p*-value of 0.1. The authors also performed ROC analysis, which indicated the satisfactory performance of salivary HO-1 in distinguishing between patients affected by PD and healthy subjects (AUC 0.86, sensitivity 83%, specificity 75%). Interestingly, even higher values were achieved for discriminating between PD patients and patients with neurodegenerative disorders (AD, MCI) or patients affected by other neurological diseases (AUC 0.87, 0.88; sensitivity 87%, 84%; specificity 84%, 76%, respectively) [55].

Additionally, Cressatti et al. [39] measured salivary HO-1 levels; however, the data were shown in comparison to miRNA, which was discussed later. Noteworthy is that all these studies used the same diagnostic and analytical methods to investigate HO-1 levels in saliva (idiopathic PD diagnosis according to the United Kingdom Parkinson’s Disease Society Brain Bank criteria and ELISA) [39,55,56]. Even though similar conclusions were presented in these papers, discrepancies regarding disease stages or different ROC results require further study.

### 4.3. MicroRNA (miRNA) and DNA

MicroRNA (miRNA) is responsible for post-transcriptional regulation of gene expression [80]. MiRNA alterations affect gene expression and, in consequence, influence various biological processes. Considering their impact and high stability in body fluids, miRNAs are considered promising biomarkers for various diseases [81,82,83].

Returning to a study by Cressatti et al. [39], the authors also investigated miR-153, miR-223, and miR-7 (a and b, both insignificant). After log transformation, levels of both molecules were significantly lower in PD patients compared to controls. In addition, in the randomly selected subsets of the study population, ratios of miR-153 or miR-223 to HO-1, t-α-syn, or o-α-syn were analyzed. Only the o-α-syn/miR-153 ratio was significantly different (increased in the PD group compared to healthy controls). Nevertheless, this did not improve test accuracy. The performance of miR-153 and miR-223 was relatively satisfactory (AUC 0.79, 0.74; sensitivity 81.8%, 72.7%; specificity 71.4%, 71.4%, respectively).

Another study presented interesting findings regarding miR-145-3p and miR-874. Expression of both molecules was significantly higher in the PD group compared to healthy participants. Importantly, both miRNAs were detected in only some participants (PD: miR-874: 14 out of 30, miR-145-3p: 16 out of 30; controls: 14 out of 30, 20 out of 30, respectively). The performed ROC curve analysis showed moderate results for miR-145-3p and miR-874 (AUC 0.707, 0.727; sensitivity 60.0%, 64.3%; specificity 75.0%, 78.6%, respectively) [62].

On the other hand, Jiang et al. [63] investigated several miRNAs, among which miR-29a-3p and miR-29c-3p were significantly downregulated, whereas miR-6756-5p was significantly upregulated in PD patients compared to healthy controls. Interestingly, some correlations were noticed in comparison of patients affected by PD, essential tremor (ET), and multiple system atrophy (MSA). The authors performed ROC analysis, which showed the best results of both miR-29 combined in distinguishing between PD patients and healthy subjects (AUC 0.773, sensitivity 66.7%, specificity 83.8%). On the other hand, miR-29a-3p alone may differentiate PD from ET and MSA.

DNA CpG methylation is correlated with gene expression and affects homeostasis and developmental processes [84]. Aberrant DNA methylation contributes to a broad spectrum of disorders, including PD [64,85]. Chuang et al. [64] focused on DNA methylation in human blood and saliva. In saliva, five CpG were found to be significantly associated with PD (cg: 24742912, 11748881, 22275276, 01820192, 15133963). Genome analysis was performed in 128 PD and 131 healthy participants.

### 4.4. Metabolomic and Proteomic Studies

Metabolomics facilitates high-throughput assessment of metabolites from biofluids, cells, tissues, or organs [86]. Proteomics opens the same possibilities concerning protein examination [87]. Recently, omics studies are rapidly evolving and seem to be the future of laboratory techniques [88]. Moreover, evidence suggests that both metabolites and proteins might be altered in PD [89,90,91].

In a metabolomic study by Kumari et al. [61], fifteen metabolites were significantly altered (increased) in PD patients compared with healthy controls (histidine, propionate, tyrosine, isoleucine, acetoin, N-acetylglutamate (NAG), acetoacetate, valine, gamma-aminobutyric acid (GABA), phenylalanine, trimethylamine-N-oxide (TMAO), acetate, alanine, fucose, and glycine). Among them, for the first eight compounds, ROC analysis provided the best results (AUC ranging 0.72–0.67, sensitivity 63.2–72.0%, specificity 59.5–67.6%). In addition, levels of butyrate metabolites significantly correlated with the H&Y stage.

On the other hand, in proteomic research by Figura et al. [60], 1328 peptides corresponding to more than 500 proteins were identified. Among them, S100-A16 protein, actin-related protein 2/3 complex subunit 1A (ARPC1A), and vacuolar protein sorting-associated protein 4B (VPS4B) were significantly lower in PD patients than healthy controls. In the ROC analysis, S100-A16 had the best performance (AUC 0.7, sensitivity 91%, specificity 67%), whereas ARPC1A and VPS4B reached worse results (AUC 0.62, 0.54; sensitivity 40%, 100%, specificity 100%, 40%, respectively).

Another proteomic study revealed eighteen compounds that differed significantly between PD and healthy participants. Cystatin SA, A, A N-acetylated, B-SSC, B S-S dimer, statherin des F43, secretory leukocyte proteinase, thymosin β4, S100A9s were significantly increased, whereas histatin 1, statherin 1P, 2P, desD1, des1-9, des1-10, proline-rich protein (PRP) 0P, 3P, and cystatin SN were significantly decreased in the PD group. Among them, cystatin B-SSC, A N-acetylated, and PRP 3P were specifically varied in PD versus non-PD subjects. Interestingly, thymosin β4 correlated positively, while statherin 2P negatively, with olfactory function and odor identification. Moreover, α-defensin 3 showed a negative correlation with the Unified Parkinson’s Disease Rating Scale (UPDRS III) [59].

### 4.5. DJ-1

DJ-1 is a small protein with oxidized forms or mutations that are associated with PD [92]. Importantly, DJ-1 exhibits neuroprotective effects; it can act as an antioxidant or an oxidative stress sensor [93]. Two studies investigated salivary DJ-1 using different analytical and diagnostic methods [57,58]. Masters et al. [58] observed significantly higher concentrations of DJ-1 in PD patients (16 individuals) compared with healthy controls (22 subjects); however, the difference disappeared after adjusting for total protein concentration. Additionally, a positive correlation between the DJ-1 level and UPDRS was noticed.

On the other hand, another research, which enrolled almost eighteen times more PD patients, did not find statistically significant alterations in DJ-1 levels. Furthermore, UPDRS was not associated with DJ-1. In turn, patients in the H&Y IV stage had higher DJ-1 concentrations compared to patients in the H&Y I–III stages or healthy subjects. Interestingly, PD patients with tremor dominant type PD or akinetic-rigid dominant type demonstrated significantly elevated DJ-1 levels compared to the mixed type [57]. Although both studies indicated DJ-1 as a possible biomarker of PD progression, discrepancies between them require further investigation to assess its reliability fully.

### 4.6. Salivary Extracellular Vesicles (sEV)

Extracellular vesicles (EV) are heterogeneous nanovesicles released by cells as functional mediators of intercellular communication [94]. EV transport biologically active molecules such as proteins, lipids, and nucleic acids. In addition, they can be found in almost all human fluids, including saliva (sEV) [95]. In recent years, sEV has attracted increasing attention as a possible diagnostic tool for multiple diseases, including cancers and neurodegenerative disorders [95,96,97].

Cao et al. [37] confirmed the presence of sEV in PD patients’ saliva. Furthermore, the authors analyzed all three forms of α-syn discussed above. The findings indicated that in sEV, both o-α-syn and o-α-syn/t-α-syn ratios were significantly elevated in the PD group compared to healthy controls, whereas p-α-syn, t-α-syn, and their ratio did not differ significantly. The ROC analysis revealed very good results of o-α-syn and satisfactory for the o-α-syn/t-α-syn ratio (AUC 0.941, 0.772; sensitivity 92%, 81%; specificity 86%, 71%, respectively).

Another study demonstrated significantly elevated sEV concentrations in the saliva of PD patients compared to healthy controls. Three different methods of Nanoparticle Tracking Analysis (NTA) were used; fluorescent-dye-tagged NTA provided the best results. Moreover, t-α-syn, CD9, CD63, flotillin-1, p-α-syn, L1CAM, and the p-α-syn/t-α-syn ratio (all in sEV) revealed significantly higher concentrations or expressions in the PD group. The ROC analysis showed excellent results in sEV performance and good t-α-syn in sEV (AUC 0.967, 0.814; sensitivity 94.3%, 88.2%; specificity 90.9%, 75.0%, respectively). Interestingly, the authors observed that prodromal PD patients had a significant increase in t-α-syn in sEV and in sEV itself, analyzed using the fluorescent-dye-tagged NTA, compared to healthy controls [43]. Although sEV seem promising, discrepancies between these two studies need further examination.

### 4.7. Alzheimer’s Disease (AD)-Related Biomarkers in PD

Interestingly, several studies analyzed AD-related biomarkers for PD: β-amyloid (Aβ) and phosphorylated or total tau (p-, t-tau) [28,98]. Bermejo-Pareja et al. [49] recruited 51 PD patients and did not reveal considerable differences in Aβ40 or Aβ42 levels between PD and healthy participants. In contrast, another study found a significant increase in Aβ42 concentrations in the PD group. The authors performed the ROC analysis, which showed relatively satisfactory results (AUC 0.77, sensitivity 91.7%, specificity 59.1%) [44]. On the other hand, Lau et al. [50] did not detect Aβ42 in the diagnostic groups.

In the same study [50], salivary p-tau and t-tau levels did not differ significantly between AD, PD, and healthy participants (each group consisted of 20 subjects). Similarly, Sabaei et al. [44] did not find significant alterations in p-tau levels between PD patients and healthy controls. Nevertheless, the ROC analysis revealed moderate results (AUC 0.64, sensitivity 91.7%, specificity 50%). No considerable changes in p-tau concentrations were also confirmed by another research, which enrolled 108 PD patients. However, in this case, t-tau was significantly elevated in the PD group compared to healthy controls. In the ROC analysis, neither t-tau nor p-tau reached significant results. Nevertheless, when the ratios of o-α-syn or t-α-syn to tau forms were investigated, the findings were more satisfactory, and the o-α-syn/t-tau ratio reached excellent results (AUC 0.963, sensitivity 92.5%, specificity 91.9%) [40]. This indicates a possible combination of o-α-syn and t-tau as a biomarker for PD; however, further study is necessary to verify this suggestion.

### 4.8. Cortisol and Lactoferrin

Lactoferrin is a glycoprotein which may exhibit neuroprotection by reducing oxidative stress and α-syn aggregation [99,100]. Carro et al. [51] investigated lactoferrin in the saliva of 59 PD patients. Interestingly, salivary lactoferrin concentrations in PD patients were significantly elevated compared to healthy controls. Surprisingly, this observation contradicts findings regarding AD reported in our previous meta-analysis [28]. Salivary lactoferrin seem to be decreased only in AD, while both in PD and other dementia such as FTD, salivary levels of lactoferrin did not decrease or even are higher than controls. A possible explanation could be associated with the regulation of innate immunity that it’s controlled by the hypothalamus region, and the hypothalamic region is differently affected in neurodegenerative disorders [101].

Cortisol is a hormone, which may alter mitochondrial function and contribute to oxidative stress or neuroinflammation, characteristic of PD [102]. One of the studies concentrated on cortisol levels in the saliva of PD patients. In a small sample (18 patients), salivary cortisol was significantly higher in PD patients than in healthy controls. Most patients affected by PD were in the H&Y II stage [52].

### 4.9. Other Proteins

Returning to a study by De Bartolo et al. [40], the authors observed significantly higher levels of both TNF-α and activated microtubule-associated protein light chain 3β (MAP-LC3β) in PD patients compared to healthy participants. The latter protein also correlated significantly with the Non-Motor Symptoms Scale (NMSS) score. In the ROC analysis, TNF-α provided satisfactory results, whereas MAP-LC3β excellent (AUC 0.660, 0.924; sensitivity 61.3%, 91.3%; specificity 90.3%, 88.7%, respectively). In addition, the authors compared ratios of t-α-syn and o-α-syn to these proteins. Magnificent results were achieved when the o-α-syn/TNF-α and o-α-syn/MAP-LC3β ratios were investigated (AUC 0.979, 0.997; sensitivity 92.5%, 100%; specificity 91.9%, 96.4%, respectively). These findings seem very promising; nevertheless, further investigation is necessary to verify the reliability of these potential biomarkers fully.

Another research investigated salivary albumin levels in PD patients. The results showed that patients had significantly elevated albumin concentrations compared to healthy controls. A significant positive correlation with DJ-1 was observed [58].

### 4.10. Other Enzymes

In a study from 2022, salivary ATP13A2 was investigated. Unfortunately, some participants had undetectable amounts of this enzyme due to the ELISA assay detection threshold. As all patients with motor complications demonstrated detectable amounts of ATP13A2, only they were further studied as the experimental group. Among detectable samples, PD patients had significantly elevated levels of salivary ATP13A2 compared to controls. Furthermore, significant correlations between ATP13A2 and total UPDRS, UPDRS III, IV, H&Y scale, and disease duration were noticed [54].

On the other hand, in a study by Fedorova et al. [53], salivary acetylcholinesterase (AChE) activity was significantly increased in PD patients compared to healthy individuals. The same observation appeared when the authors analyzed the AChE activity/total salivary protein ratio. Moreover, statistically significant correlations were observed between AChE activity and H&Y stages, which might reflect PD progression.

Another research investigated salivary amylase levels in PD patients. The results indicated that the experimental group had significantly higher amylase levels compared with healthy subjects. Moreover, amylase positively and significantly correlated with DJ-1 [58].

### 4.11. Study Limitations

Among the main limitations is the heterogeneity of the included studies in terms of the recruitment criteria, demographic characteristics of the subjects and the methods of laboratory diagnostics. The impact of factors such as duration and progression of PD, and diagnosis based primarily on clinical criteria should also be highlighted. The potential causes of bias were lack of randomization or blinding of participants, as well as not specified sample size justification. Most studies present only comparisons instead of ROC analysis, which is dedicated to predictive usability assessment. Also, due to the variety of markers, it was difficult to compare their usefulness in clinical diagnostics.

## 5. Conclusions

In conclusion, some potential biomarkers, especially alpha-synuclein, could be significantly altered in the saliva of patients with Parkinson’s Disease. Despite variability in salivary alpha-synuclein levels due to heterogeneity of enrolled patients, combining the molecular panel with other relevant markers should be considered. However, further studies are necessary to confirm these findings about reliable usefulness for early diagnosis of neurodegenerative diseases and investigate potential utility for differential diagnosis with other synucleopathies.

## Figures and Tables

**Table 1:** Inclusion and exclusion criteria according to the PI(E)COS.

Parameter	Inclusion Criteria	Exclusion Criteria
Population	Patients aged 0–99 years, both genders; sample size: 15 patients or more	sample size: below 15 patients or controls
Intervention/Exposure	Parkinson’s Disease	Other diseases, e.g., secondary parkinsonism
Comparison	Not exposed control group	Lack of control group
Outcomes	Alterations in salivary markers level	Alterations in other markers level (e.g., serum), microbiota
Study design	Case–control, cohort, and cross-sectional studies	Literature reviews, case reports, expert opinion, letters to the editor, conference reports
	Published after 1 January 2008	Not published in English

**Table 2:** The characteristics of included studies.

Author, Year	Setting	Study Group (F/M), Age	Control Group (F/M), Age	Type of Saliva and Method of Collection	Centrifugation and Storing	Method of Marker Determination	Salivary Biomarkers
Al-Nimer et al., 2014 [35]	Iraq	PD: 20 (4/16), 64.4 ± 10.6 (66)	20 (2/18), 65.4 ± 8.2 (64)	unstimulated saliva collected into disposable containers	centrifuged at 3000 rpm for 10 min, stored at −20 °C	ELISA	t-α-syn
Angius et al., 2023 [36]	Italy	PD: 15 (5/10), 74.7 ± 7.1	23 (11/13), 73.9 ± 6.6	3 mL of saliva collected by drooling into a 50 mL vial	immediately placed on ice, centrifuged twice for 15 min at 4 °C (2600× *g* and 15,000× *g*, respectively), stored at −80 °C	ELISA	t-α-syn (ns), p-α-syn (ns), o-α-syn
Cao et al., 2019 [37]	China	PD: 74 (34/40), 59.62 ± 8.57	60 (34/26), 58.75 ± 9.85	unstimulated saliva collected by drooling between 9 and 11 a.m.	immediately placed on ice, precleared by a low spin at 2600× *g* for 15 min at 4 °C, and at 15,000× *g* for 15 min at 4 °C, stored at −80 °C	electrochemiluminescence (ECL) immunoassays	in sEV: t-α-syn, p-α-syn (ns), o-α-syn
Chahine et al., 2020 [38]	North America	PD: 59 (18/41), 63.1 ± 8.6	21 (12/9), 61.0 ± 6.3	5 mL of unstimulated whole saliva	centrifuged at 2000× *g* for 15 min at 4 °C, stored −80 °C	ELISA	t-α-syn (ns)
Cressatti et al., 2020 [39]	Canada	PD: 84 (35/49), 71.39 (1.38)	83 (44/39), 67.31 (1.04)	whole unstimulated saliva collected by passive drooling	centrifuged at 10,000 rpm for 20 min at 4 °C, stored at −80 °C	ELISA, RT-qPCR	t-α-syn (ns), o-α-syn (ns), HO-1 (ns), miR-153, miR-223, miR-7a (ns), miR-7b (ns)
De Bartolo et al., 2023 [40]	Italy	PD: 1st cohort: 80 (25/55), 64.5 ± 9; 2nd cohort: 28 (13/20), 62 ± 11	1st cohort: 62 (sex and age matched); 2nd cohort: 28 (sex and age matched)	3 mL of saliva collected by drooling into a 50 mL vial	immediately placed on ice, centrifuged at 5000× *g* for 20 min at 4 °C, stored at −80 °C	ELISA	t-α-syn (ns), o-α-syn, t-tau, p-tau (ns), MAP-LC3β, TNF-α
Fernández-Espejo et al., 2021 [41]	Spain	PD: 45 (18/27), 61.4 ± 18.5	30 (18/12), 59.6 ± 11	3 mL of saliva collected into 5 mL polypropylene tubes	centrifuged at 2500 rpm for 10 min, immediately frozen and stored at −80 °C	ELISA	t-α-syn (ns), 3-nitrotyrosine proteins (ns)
Goldman et al., 2018 [42]	USA	PD: 115 (43/72), 68.24 (6.40)	88 (43/45), 68.24 (6.40)	collected in the morning	NR	ELISA	t-α-syn (ns)
Rastogi et al., 2023 [43]	India	PD: 70 (NR), 56.2 (30–79); prodromal PD: 8 (NR), 58.25 (52–75)	26 (NR), 55.0 (40–75)	2 mL of unstimulated saliva collected from the floor of the mouth	kept on ice, centrifuged at 1700× *g* for 20 min at 4 °C and at 10,000× *g* for 20 min at 4 °C, kept at 4 °C for further experiments, stored at −80 °C for longer period	fluorescence (lipid-binding dye-labeled) NTA, antibody-based (CD63 Alexa fluor 488 tagged sEV) NTA, scatter-based NTA, Western Blot, ELISA	sEV, in sEV: t-α-syn, CD9, CD63, flotillin-1, p-α-syn, L1CAM
Sabaei et al., 2023 [44]	Iran	PD: 24 (10/14), 61.2 ± 8.7; AD: 24 (10/14), 73.5 ± 9.8	22 (13/9), 64.1 ± 9.2	dental cotton roll placed on the oral side of the cheek, moist rolls located inside the salivary collector tubes	centrifuged at 1500 rpm for 5 min, stored at −80 °C	ELISA	Aβ42, p-tau (ns), t-α-syn
Shaheen et al., 2020 [45]	Egypt	PD: 25 (10/15), 60.1 ± 5.6	15 (5/10), 60 ± 6.7	3 mL of saliva collected by drooling into a 50 mL vial	immediately placed on ice, centrifuged at 2600× *g* for 15 min at 4 °C and at 15,000× *g* for 15 min at 4 °C, stored at −80 °C	ELISA	t-α-syn, o-α-syn
Kang et al., 2016 [46]	China	PD: 201 (79/122), 63.18 ± 9.67	67 (26/41), 61.04 ± 10.01	unstimulated saliva collected between 9 and 11 a.m. into a 15 mL pre-chilled vial	immediately placed on ice, centrifuged at 2600× *g* for 15 min at 4 °C, and at 15,000× *g* for 15 min at 4 °C, stored at −80 °C	Luminex assay	t-α-syn (ns), o-α-syn
Vivacqua et al., 2016 [47]	Italy	PD: 60 (29/31), 66.3 ± 8.78	40 (18/22), 68.3 ± 7.9	3 mL of saliva collected by drooling into a 50 mL vial	immediately placed on ice, centrifuged at 2600× *g* for 15 min at 4 °C and at 15,000× *g* for 15 min at 4 °C, stored at −80 °C	ELISA	o-α-syn, t-α-syn
Vivacqua et al., 2019 [48]	Italy	PD: 112 (53/59), 69.01 ± 11.16; PSP: 22 (10/12), 68.84 ± 6.16	90 (37/53), 62.09 ± 15.08	1 mL of saliva collected by drooling into a 50 mL vial	immediately placed on ice, centrifuged at 10,000× *g* for 10 min at 4 °C, stored at −80 °C	ELISA	o-α-syn, t-α-syn
Bermejo-Pareja et al., 2010 [49]	Spain	PD: 51 (25/26); 72.96 (60–93); AD: 70 (49/21), 77.20 (60–91)	56 (39/17), 74.35 (64–85)	approx. 1 mL of saliva collected at around 1 a.m. in sterile plastic containers previously treated with 2% sodium azide solution	centrifuged at 1500 rpm for 5 min, immediately frozen at −80 °C until used	ELISA	Aβ42 (ns), Aβ40 (ns)
Lau et al., 2015 [50]	Korea	PD: 20 (11/9), 73 ± 8.07; AD: 20 (12/8), 72.5 ± 7.68	20 (15/5), 66.1 ± 7.79	3 mL unstimulated saliva collected by spitting	centrifuged at 1000× *g* for 15 min, stored at −80 °C	ELISA, EG-ISFET	Aβ42 (not detected), p-tau (ns), t-tau (ns), trehalose (ns)
Carro et al., 2017 [51]	Spain	PD: 59 (32/27), 69.5 ± 8.6; AD: 80 (49/31), 76.2 ± 5.33; MCI: 44 (25/19), 75.16 ± 5.13	91 (59/32), 73.7 ± 6.88	0.5 mL of unstimulated whole saliva collected into sterile plastic containers precoated with 2% sodium azide solution	immediately placed on ice, precleared by a low spin at 600× *g* for 10 min at 4 °C, stored at −80 °C	ELISA	lactoferrin
Costa et al., 2019 [52]	Brazil	PD: 18 (6/12), 68 (62.5–71.5)	17 (7/10), 62 (60–66)	collected in the morning with a piece of cotton, placed under the tongue	centrifuged, stored at −20 °C	ELISA	cortisol
Fedorova et al., 2015 [53]	Denmark	PD: 30 (14/16), 63.7 ± 9.1	49 (22/27), 62.7 ± 9.4	collected by spitting into a pre-weighted test tube, saliva collected during the first 5 min was discarded, saliva obtained during the following 10–50 min was analyzed	immediately placed on ice, centrifuged at 3000 rpm for 30 min, stored at −80 °C	colorimetric method	AChE
Fernández-Espejo et al., 2022 [54]	Spain	PD: 64 (31/33), 65.5 ± 11.7	32 (14/18), 61.4 ± 10	3 mL of saliva collected into 5 mL polypropylene tubes	centrifuged at 2500 rpm for 10 min, immediately frozen and stored at −80 °C	ELISA	ATP13A2
Galindez et al., 2021 [55]	Canada	PD: 75 (18/57), 72.65 ± 11	162 (99/63), 62.19 ± 12	unstimulated whole saliva collected by passive drooling	kept at 4 °C for a maximum of 3 h, centrifuged at 10,000 rpm for 20 min at 4 °C, stored at −80 °C	ELISA	HO-1
Song et al., 2018 [56]	Canada	PD: 58 (28/30), 70.83 ± 7.85	59 (28/31), 66.74 ± 7.63	collected by spitting into sterilized centrifuge tubes	kept at 4 °C for a maximum of 3 h, centrifuged at 10,000× *g* for 20 min at 4 °C, stored at −80 °C	ELISA	HO-1
Kang et al., 2014 [57]	China	PD: 285 (114/171), 63.34 ± 9.11	91 (32/59), 61.59 ± 10.61	unstimulated saliva collected between 9 and 11 a.m. into a 15 mL pre-chilled vial	kept in the ice, centrifuged at 2600 × *g* for 15 min at 4 °C, and at 15,000× *g* for 15 min at 4 °C, stored at −80 °C	Luminex assay	DJ-1
Masters et al., 2015 [58]	UK	PD: 16 (3/13), 61 ± 12	22 (11/11), 62 ± 16	unstimulated whole saliva collected by passive drooling into a pre-weighed sterile 20 mL tube	centrifuged at 16,300 × *g* for 5 min	quantitative immunoblotting, amylase activity assay, ELISA, periodic-acid Schiff staining of SDS-gels	DJ-1, amylase, mucin (ns), albumin
Contini et al., 2023 [59]	Italy	PD: 36 (11/15), 72 ± 7; AD: 35 (23/12), 80 ± 6	36 (18/18), 78 ± 6	unstimulated whole saliva collected between 9 and 12 a.m. with a soft plastic aspirator for less than 1 min, transferred to a plastic tube cooled on ice	centrifuged at 20,000× *g* for 15 min at 4 °C, stored at −80 °C or immediately analyzed	RP-HPLC-LR-ESI-MS analysis	proteomics
Figura et al., 2021 [60]	Poland	PD: 24 (9/15), 61.6 ± 8.2	15 (5/9), 60.9 ± 6.7	collected in the morning using RNA-Pro-Sal kits	immediately frozen at −80 °C	LC-MS/MS mass spectrometry	proteomics
Kumari et al., 2020 [61]	India	PD: 76 (17/59), 54.96 ± 7.82	37 (23/14), 53 ± 8.57	2 mL of unstimulated whole saliva collected by swab (passive drooling) between 9 and 11 a.m.	immediately stored at −80 °C, centrifuged at 2000 × *g* for 10 min at 4 °C	NMR	metabolomics
Chen et al., 2020 [62]	China	PD: 30 (10/20), 63.20 ± 10.17	30 (14/16), 59.57 ± 12.83	collected at a fasting state in the morning	centrifuged at 3000× *g* for 15 min at 4 °C and at 12,000 × *g* for 10 min at 4 °C, stored at −80 °C	RT-qPCR	miR-874, miR-145-3p
Jiang et al., 2021 [63]	China	PD: 50 (31/19), 63.62 ± 11.65	30 (16/14), 59.67 ± 11.18	1–3 mL of saliva collected	kept at 4 °C for a maximum of 3 h, centrifuged at 12,000× *g* for 20 min at 4 °C, stored at −80 °C	RT-qPCR	miR-29a-3p, miR-29c-3p, miR-6085 (ns), miR6724-5p (ns), miR-6893-5p (ns), miR-6756-5p, miR-6892-3p (ns), miR4731-3p (ns)
Chuang et al., 2017 [64]	USA	PD: 128 (NR), NR	131 (sex and age matched	NR	NR	Illumina HumanMethylation450 BeadChip	DNA methylation

Legend: Aβ, beta-amyloid; AChE, acetylcholinesterase; AD, Alzheimer’s Disease; ATP, adenosine triphosphate; CD, cluster of differentiation; EG-ISFET, extended gate ion-sensitive field-effect transistor; ELISA, enzyme-linked immunosorbent assay; HO-1, heme oxygenase-1; LC-MS/MS, liquid chromatography tandem mass spectrometry; L1CAM, L1 cell adhesion molecule; MAP-LC3β, microtubule-associated protein light chain 3 beta; miR, microRNA; NMR, nuclear magnetic resonance; NR, not reported; ns, not significant; NTA, nanoparticle tracking analysis; o-α-syn, oligomeric-alpha-synuclein; PD, Parkinson’s Disease; p-α-syn, phosphorylated-alpha-synuclein; PSP, progressive supranuclear palsy; p-tau, phosphorylated tau; RT-qPCR, reverse transcription quantitative polymerase chain reaction; RP-HPLC-LR-ESI-MS, reversed-phase high-performance liquid chromatography with low-resolution electrospray ionization mass spectrometry; sEV, small extracellular vesicles; TNF-α, tumor necrosis factor-alpha; t-α-syn, total-alpha-synuclein; t-tau, total tau; UK, the United Kingdom; USA, the United States of America.

**Table 3:** Reported predictive parameters of most discriminant potential biomarkers for Parkinson’s Disease (vs. healthy controls) from included studies.

Study	Most Discriminant Markers	AUC	−95% CI	+95% CI	Sensitivity [%]	Specificity [%]
Cao et al., 2019 [37]	o-α-syn in sEV	0.941	0.896	0.985	92	86
Chen et al., 2020 [62]	miR-874	0.727	-	-	64.3	78.6
	miR-145-3p	0.707	-	-	60	75
Cressatti et al., 2020 [39]	miR-153	0.79	64.5	99.2	81.8	71.4
	miR-223	0.74	59.6	93.0	72.7	71.4
De Bartolo et al., 2023 [40]	o-α-syn	0.998	-	-	100	98.39
	MAP-LC3β	0.924	-	-	91.25	88.71
	TNF-α	0.660	-	-	61.25	90.32
Figura et al., 2021 [60]	S100A16	0.7	-	-	91	67
	ARPC1A	0.62	-	-	40	100
Galindez et al., 2021 [55]	HO-1	0.86	0.81	0.91	83	75
Jiang et al., 2021 [63]	miR-29a-3p	0.692	0.573	0.812	79.3	51.2
	miR-29c-3p	0.722	0.583	0.861	65.4	70.6
	miR-6756-5p	0.640	0.505	0.774	66.7	58.6
	miR-29a-3p and miR-29c-3p (combined)	0.773	0.639	0.908	66.7	83.8
Kumari et al., 2020 [61]	histidine	0.72	0.61	0.80	64.00	64.86
	propionate	0.71	0.60	0.80	68.42	67.57
	tyrosine	0.69	0.59	0.79	72.00	59.46
	isoleucine	0.69	0.58	0.78	65.79	67.57
	acetoin	0.68	0.57	0.77	63.16	62.16
	NAG	0.67	0.56	0.76	65.79	59.46
	acetoacetate	0.67	0.56	0.77	64.86	64.86
	valine	0.67	0.56	0.76	67.11	64.86
Rastogi et al., 2023 [43]	sEV	0.967	-	-	94.34	90.91
	t-α-syn in sEV	0.814	-	-	88.24	75.00
Sabaei et al., 2023 [44]	Aβ42	0.77	-	-	91.7	59.1
	t-α-syn	0.68	-	-	95.8	36.4
	p-tau	0.64	-	-	91.7	50.0
Shaheen et al., 2020 [45]	t-α-syn	0.823	-	-	80.0	86.7
	o-α-syn	0.724	-	-	76.0	60.0
Song et al., 2018 [56]	HO-1	0.76	0.63	0.90	75	70

Legend: Aβ, beta-amyloid; HO-1, heme oxygenase-1; MAP-LC3β, microtubule-associated protein light chain 3 beta; miR, microRNA; NAG, N-acetylglutamate; o-α-syn, oligomeric-alpha-synuclein; p-α-syn, phosphorylated-alpha-synuclein; p-tau, phosphorylated tau; sEV, small extracellular vesicles; TNF-α, tumor necrosis factor-alpha; t-α-syn, total-alpha-synuclein; t-tau, total tau; -, not reported.

**Table 4:** Detailed results for meta-analysis comparing salivary levels of alpha-synuclein for Parkinson’s Disease vs. healthy controls.

Study	SMD	95% CI	*p*-Value	Weight
Alpha-synuclein total				
Al-Nimer et al., 2014 [35]	−0.786	−1.438 to −0.134		7.62
Angius et al., 2023 [36]	−0.172	−0.832 to 0.488		7.59
Cao et al., 2019 [37]	−0.024	−0.366 to 0.317		8.99
Chahine et al., 2020 [38]	0.025	−0.476 to 0.526		8.31
De Bartolo et al., 2023 [40]	0.050	−0.283 to 0.383		9.02
Fernández-Espejo et al., 2021 [41]	−0.112	−0.578 to 0.353		8.48
Goldman et al., 2018 [42]	0.349	−0.229 to 0.927		7.97
Kang et al., 2016 [46]	−0.027	−0.303 to 0.250		9.22
Sabaei et al., 2023 [44]	−0.771	−1.378 to −0.164		7.83
Shaheen et al., 2020 [45]	−1.106	−1.800 to −0.411		7.41
Vivacqua et al., 2016 [47]	−1.817	−2.293 to −1.341		8.42
Vivacqua et al., 2019 [48]	−1.203	−1.505 to −0.900		9.13
Total (random effects)	−0.458	−0.835 to −0.081	0.017	
Alpha-synuclein oligomeric				
Angius et al., 2023 [36]	0.879	0.189 to 1.568		18.49
De Bartolo et al., 2023 [40]	1.406	1.034 to 1.777		21.14
Shaheen et al., 2020 [45]	0.772	0.101 to 1.443		18.66
Vivacqua et al., 2016 [47]	2.304	1.788 to 2.820		19.99
Vivacqua et al., 2019 [48]	0.462	0.180 to 0.744		21.72
Total (random effects)	1.165	0.488 to 1.841	0.001	

## Data Availability

Data are available on request from the corresponding author.

## Supplementary Materials

The following supporting information can be downloaded at: https://www.mdpi.com/article/10.3390/cells13040340/s1, Table S1: The detailed characteristics of included studies.
