# Supplementary material for: Salivary Biomarkers for Parkinson’s Disease: A Systematic Review with Meta-Analysis
Source: Cells. 2024 Feb 14;13(4):340. doi: 10.3390/cells13040340 (PMC10887027; doi:10.3390/cells13040340)
Supplement: Supplementary file 1 [file cells-13-00340-s001.zip › cells-2855901-supplementary.pdf]

Nijakowski et al. „Salivary biomarkers for Parkinson's Disease: a systematic review with meta-analysis" – Supplementary materials

Table S1. The detailed characteristics of included studies.

Study	Inclusion Criteria	Exclusion Criteria	Smoking Status
Al-Nimer et al., 2014	PD: PD (primary, familial, and sporadic), different duration of illness, treated with dopamine agonists and/or anticholinergic agents, aged 40-75 years	HC: severely debilitated, aged more than 80 or less than 15 years	current smokers: 4 PD, 10 HC; ex-smokers: 3 PD, 4 HC
Angius et al., 2023	NR	PD: atypical parkinsonism, dementia, psychiatric conditions interfering with study participation; HC: history of any neurodegenerative disorder	NR
Cao et al., 2019	PD: PD diagnosis according to the United Kingdom Parkinson's Disease Society Brain Bank criteria	all: cardiovascular, cerebrovascular, autoimmune diseases, hematological neoplasms and tumors	NR
Chahine et al., 2020	PD: clinical diagnosis of PD, presence of bradykinesia with either rest tremor or rigidity; decreased dopamine transporter (DAT) binding on SPECT, age ≥40 years, any stage of disease; HC: normal DAT SPECT	all: clinical diagnosis of dementia, comorbid medical conditions precluding specimen acquisition	NR
Cressatti et al., 2020	PD: idiopathic PD diagnosis according to the United Kingdom Parkinson's Disease Society Brain Bank criteria	all: cigarette smoking within the past year, history of oral cancer, severe periodontal disease, active systemic inflammatory disease, current alcoholism, illicit drug abuse, recent exposure to neuroleptic medications, atypical, secondary, or familial parkinsonism	non-smokers
De Bartolo et al., 2023	PD: PD diagnosis according to Vivacqua et al. and Ohtomo et al. criteria for less than 2 years	PD: other neurological or psychiatric conditions, H&Y stage III, IV, V; dopaminergic treatment; HC: neurological or psychiatric disorders, drugs inducing parkinsonism, related to a parkinsonian patient; all: cardiovascular diseases, diabetes mellitus, chronic inflammatory diseases, neoplasms, autoimmune diseases, salivary gland/oral cavity pathologies	NR
Fernández-Espejo et al., 2021	PD: PD diagnosis according to MDS criteria, a reliable loss of dopamine-transporter binding signal on basal ganglia (evaluated with DAT-SPECT), aged 45-75 years on onset, PD diagnosis for at least 3 years	PD: atypical deficits, relatives with PD; HC: first-degree family member with a neurological disorder; all: liver, renal, hematological, cardiovascular dysfunctions, AIDS, autoimmune diseases, cancer, alcoholism, smoking, coffee drinking	non-smokers
Goldman et al., 2018	PD: PD diagnosis according to the United Kingdom Parkinson's Disease Society Brain Bank criteria, moderate-advanced	all: atypical, secondary parkinsonian syndromes, Deep Brain Stimulation, ablative brain surgery, cancer (except basal or	NR

	stage, disease duration at least 4 years, onset at 50-75 years, bradykinesia, rigidity, resting tremor, well-established response to dopaminergic agents, amantadine	squamous cell skin cancers) within preceding 5 years, autoimmune, liver, hematological disorders, conditions precluding lumbar puncture; HC: neurological disorders, MoCA less than 26, first-degree family member with PD	
Rastogi et al., 2023	PD: clinical definite diagnosis of PD	NR	NR
Sabaei et al., 2023	PD: PD diagnosis according to MDS 2015 criteria, PD: MMSE score ≥ 26, H&Y stage I or II; HC: MMSE score equal to or more than 26, no history of neurological diseases; AD: AD diagnosis according to the NIA-AA criteria, no other diseases that affect cognition	AD, PD: drugs other than medications for treating the underlying neurological disease; all: history of vascular dementia, head injury, stroke, liver or kidney failure, other dementias	NR
Shaheen et al., 2020	PD: idiopathic PD diagnosis according to the United Kingdom Parkinson's Disease Society Brain Bank criteria	all: cardiovascular, cerebrovascular, autoimmune, chronic inflammatory diseases, diabetes mellitus, hematological neoplasms, salivary gland, oral cavity pathologies	NR
Kang et al., 2016	PD: PD diagnosis according to the United Kingdom Parkinson's Disease Society Brain Bank criteria	PD: functional neurosurgery for PD	NR
Vivacqua et al., 2016	PD: PD diagnosis based on Queen Square Brain Bank criteria, MoCA score higher than 18, FAB score higher than 12	PD: atypical, secondary parkinsonisms; all: cardiovascular, cerebrovascular, autoimmune, chronic inflammatory diseases, hematological neoplasms, diabetes mellitus, solid tumors, salivary gland and oral cavity pathologies	NR
Vivacqua et al., 2019	PD: PD diagnosis based on Queen Square Brain Bank criteria	all: cardiovascular, cerebrovascular, autoimmune, chronic systemic diseases, hematological and solid neoplasms, salivary gland and oral cavity pathologies, MoCA score lower than 18, FAB score lower than 12	NR
Bermejo-Pareja et al., 2010	PD: PD diagnosis according to Gelb et al. and Calne et al. diagnostic criteria; AD: AD diagnosis according to the DSM-IV or NINCDS-ADRDA criteria; HC: completely normal cognitive and functional level	AD: vascular dementia	NR
Lau et al., 2015	PD: PD diagnosis; AD: AD diagnosis; HC: at least 50 years of age, good general health; series of clinical and neuropsychological	HC: history of neurological, psychiatric, major medical diagnosis that could contribute significantly to cognitive impairment or dementia	PD: 2 smokers, 4 ex-smokers; AD: 3 smokers, 1 ex-smoker; HC: 3

	examinations by physicians and neuropsychologists defined participants' inclusion to appropriate groups		smokers, 1 ex-smoker
Carro et al., 2017	PD: PD diagnosis according to Gelb et al. and Calne et al. diagnostic criteria; HC: CDR scale = 0, MMSE score = 29 (0.8), normal yields in cognitive tests of memory, language, executive functions; AD: AD diagnosis according to the NINCDS-ADRDA criteria; MCI: MCI diagnosis based on the Petersen criteria	HC: psychiatric, systemic or neurological disorders that can affect cognition or limit participation in the study, visual, auditory, language limitations	NR
Costa et al., 2019	PD: PD diagnosis for at least 2 years, H&Y score greater than or equal to 1.5, no regular physical activity (twice a week or more), age 60 years or more, pharmacological stability (unchanged drug prescription for at least 3 months), use of levodopa, completion of the TCLE	PD: polyneuropathy, other neurodegenerative pathology, autoimmune diseases, cognitive deficit incompatible with study participation, glucocorticoids, corticosteroids, insulin, antiepileptic drugs, drugs for dementia used 2 weeks prior to data collection, active malignancy, history of neurosurgical procedure; HC: regular physical activity, corticosteroids, any ongoing disease	NR
Fedorova et al., 2015	PD: PD diagnosis according to the United Kingdom Parkinson's Disease Society Brain Bank criteria	PD: serious medical conditions (severe disorders of the heart, lungs, liver, and kidneys), neurological disorders, diseases affecting the autonomic function or the salivary glands, cancer or radiation therapy of the head and neck, drugs that may affect the function of AChE	NR
Fernández-Espejo et al., 2022	PD: PD diagnosis according to MDS criteria, a reliable loss of dopamine-transporter binding signal on basal ganglia (evaluated with DAT-SPECT), aged 45-75 years on onset	PD: atypical deficits, Deep Brain Stimulation, first-degree relatives with PD; HC: first-degree family member with a neurological disorder; all: liver, renal, hematological, cardiovascular dysfunctions, malabsorption, morbid obesity, acquired immunodeficiency syndrome, autoimmune diseases, cancer, infectious conditions, alcoholism, smoking, coffee drinking	non-smokers
Galindez et al., 2021	PD: idiopathic PD diagnosis according to the United Kingdom Parkinson's Disease Society Brain Bank criteria; other participants: based on their neurological status	all: cigarette smoking within the past year, active systemic inflammatory disease, history of oral cancer, current alcoholism or drug abuse, atypical or familial parkinsonism	non-smokers
Song et al., 2018	PD: idiopathic PD diagnosis according to the United	all: atypical or secondary parkinsonism, smoking, severe	non-smokers

	Kingdom Parkinson's Disease Society Brain Bank criteria	periodontal disease, active systemic inflammatory diseases, oral cancer, HC: history of a neurological disorder	
Kang et al., 2014	PD: PD diagnosis according to the United Kingdom Parkinson's Disease Society Brain Bank criteria	all: moderate, heavy cigarette smoking (more than 10 packs/year), alcohol use, psychotherapeutic drugs; HC: history, signs, symptoms of Parkinson-plus syndrome, cognitive impairment, other neurological diseases	smoking ≤10 packs/year
Masters et al., 2015	PD: PD diagnosis based on Queen Square Brain Bank criteria	HC: dopaminergic medication	NR
Contini et al., 2023	PD: PD diagnosis according to MDS 2015 criteria; AD: AD diagnosis according to the NIA-AA criteria	all: major oral disease (periodontitis, caries, dry mouth), history of radiotherapy, chemotherapy, antidepressants, anticholinergic drugs; PD: secondary parkinsonism, atypical Parkinsonian disorders, dementia, psychiatric conditions interfering with study participation, chronic/acute rhinosinusitis, any systemic disease with smell disorders, chronic renal failure, thyroid disorders	NR
Figura et al., 2021	PD: PD diagnosis according to the United Kingdom Parkinson's Disease Society Brain Bank criteria	all: smoking, previous injection of botulinum toxin to salivary glands, anticholinergic medications, other neurodegenerative diseases than PD, malignancies, periodontitis, active inflammation, oral cancer history; HC: clinical evidence of neurodegeneration	non-smokers
Kumari et al., 2020	PD: idiopathic PD diagnosis according to the United Kingdom Parkinson's Disease Society Brain Bank criteria	PD: secondary Parkinsonism, other neurological disorder, diabetes, hypertension; HC: neurological, psychiatric problems, diabetes, hypertension	PD: 7 smokers, 69 non-smokers; HC: 3 smokers, 31 non-smokers
Chen et al., 2020	PD: PD diagnosis based on Chinese diagnostic criteria for PD (2016)	all: diseases affecting the secretion of saliva, connective tissue diseases, severe diseases of the heart or lungs, tumor lesions, hemorrhagic wounds in the mouth, neuropsychiatric disorders, intolerance to saliva collection	NR
Jiang et al., 2021	PD: idiopathic PD diagnosis according to MDS 2015 criteria; other disorders diagnosed according to appropriate criteria	all: severe periodontal disease, oral cancer, gastroenteric tumor, active systemic inflammatory diseases, serious organic diseases of heart, liver, kidney, drug, alcoholic abuse in the past year	NR

Chuang et al., 2017	PD: PD diagnosis according to UK Brain Bank and Gelb et al. diagnostic criteria for diagnosing idiopathic PD; HC: according to previously described criteria	NR	116 smokers, 143 non-smokers

Legend: AChE, acetylcholinesterase; AD, Alzheimer's Disease; CDR, Clinical Dementia Rating; DSM, Diagnostic and Statistical Manual of Mental Disorders; FAB, Frontal Assessment Battery; HC, healthy control; H&Y, Hoehn and Yahr; MCI, Mild Cognitive Impairment; MDS, Myelodysplastic Syndromes; MMSE, Mini-Mental State Examination; MoCA, Montreal Cognitive Assessment; NIA-AA, National Institute on Aging and Alzheimer's Association; NINCDS-ADRDA, National Institute of Neurological and Communicative Disorders and Stroke and the Alzheimer's Disease and Related Disorders Association; NR, not reported; PD, Parkinson's Disease; SPECT, single photon emission computed tomography; TCLE, Total Clinical Load of Epilepsy; UK, United Kingdom.
